# Effects of Dietary Supplements on Iron-Loading Susceptibility Artefacts in Pelvic MRI

**DOI:** 10.7759/cureus.65605

**Published:** 2024-07-28

**Authors:** Justin Samuels, Jarad Martin, Matthew Richardson, Kate Skehan

**Affiliations:** 1 Radiation Oncology, Calvary Mater Hospital, Newcastle, AUS

**Keywords:** dietary supplements, curcumin affecting pelvic mri, iron accumulation susceptibility, susceptibility artefact, mri simulation, complementary medicine, mri artefact, pelvic mri

## Abstract

We present a case of an 80-year-old male who attended an MRI scan for his prostate cancer radiotherapy planning. His safety screening did not identify any contraindications to our department's MRI safety policy; however, his MRI images displayed significant susceptibility artefacts in the sigmoid colon and rectum and were not clinically acceptable. Further history revealed he had begun regularly taking curcumin supplements at the time of his prostate cancer diagnosis. The patient was instructed to cease taking the curcumin supplements and a repeat MRI appointment was scheduled for one week later. After discontinuing curcumin, repeat imaging was artefact-free and suitable for radiotherapy planning.

The chelating properties of curcumin could potentially lead to an accumulation of iron in the bowel, causing MRI susceptibility artefacts in pelvic scans and presenting a possible negative impact on the clinical utility of the images. It may be helpful to screen regular medications including health supplements with known chelation properties where MRI scan quality may be affected.

## Introduction

In 2022, prostate cancer was the most commonly diagnosed non-cutaneous cancer for Australian males, and the most commonly diagnosed cancer type in Australia overall [1].

External beam radiation therapy (EBRT) is a commonly used treatment modality for prostate cancer [2] and relies heavily on precise targeting of the gross tumour volume (GTV) and limiting the radiation exposure of nearby organs at risk (OAR). Imaging methods, including computed tomography (CT) and magnetic resonance imaging (MRI), are crucial to accurate volume identification and patient positioning during the radiotherapy planning (RTP) process [3].

CT simulation provides detailed anatomical and spatial information with the patient immobilised in the same position used during radiotherapy treatment, MRI simulation further complements this by offering high-resolution soft tissue images that are fused with CT for RTP [4].

MRI simulation in the pelvic region involves collecting multiple sequences with differing tissue weightings to account for tissue composition differences and to aid disease and OAR visualisation. Prior to attending prostate RTP appointments, patients are instructed to adhere to our departmental preparatory bladder and bowel protocol which lists food and drinks to avoid such as beans or carbonated beverages, as well as suggested volumes of water to drink prior to attending simulation. Adherence to this preparatory bladder and bowel protocol ensures that a comfortably full bladder and rectum devoid of faeces and flatus is achieved, with the goal of creating optimal anatomical conditions for OAR sparing and safe dose delivery to the planning target volume (PTV). Appointment scheduling reflects the necessary time to prepare, safety screen and acquire prostate RTP sequences. Following scan acquisition, image quality is examined by the MRI staff and at times image optimisation or repeat scanning is required. If scan quality is acceptable, the images are exported to the treatment planning software (TPS) to be utilised for radiation oncologist (RO) contouring and treatment plan generation. The CT and MRI are fused together in the TPS, allowing both imaging modalities to be viewed simultaneously by the RO whilst defining the GTV and allowing treatment planning optimisation. If the scan quality is unacceptable and unable to be rectified, the scan is aborted, and the RO is notified.

We report on a case where an MRI simulation for prostate cancer generated unacceptable images demonstrating significant magnetic susceptibility artefact in the small bowel, sigmoid colon and rectum; a hypothesis of curcumin supplements causing the artefact through iron-accumulation in the faeces was put forward, corrective action taken, and successful image acquisition obtained thereafter. To our knowledge, there are no other papers that have reported a similar clinical situation and demonstrated comparable findings.

## Case presentation

An 80-year-old male diagnosed with high-risk prostate cancer (iPSA 6.35, Grade group 5, Stage T2CN0M0) was prescribed a long course of androgen deprivation therapy (ADT) in conjunction with prostate and pelvic node EBRT. The patient had three fiducial markers inserted into the prostate by a urologist and had commenced ADT approximately one month before his scheduled simulation appointment at our radiation oncology department. Our in-house standard imaging protocol for prostate radiotherapy simulation consists of a CT scan followed by an MRI scan in the planned treatment position. Standard pre-planning workup of patient documentation and history revealed no contraindications to scanning with either modality.

Prior to admittance into the MRI scanning room, a safety questionnaire was completed with the patient, further assessing their relevant medical history and determining if any non-disclosed scanning contraindications existed. Primarily, the safety questionnaire considered factors such as previous surgeries, implantable medical devices, the possibility of metallic foreign bodies, and larger body habitus considerations that may physically exceed the MRI simulator bore aperture size of 70cm.

According to our department's MRI safety policy, the patient did not exhibit any scanning contraindications and was permitted entry to our dedicated 3-Tesla MRI Simulator (MAGNETOM Skyra, Siemens Healthineers, Erlangen, Germany) for routine prostate scanning, adhering to departmental protocol with standard acquisition parameters for the sequences to be acquired for RTP (Table 1).

**Table 1:** Prostate radiotherapy MRI acquisition parameters SPACE: Sampling Perfection with Application optimised Contrast using different flip angle Evolution; GRE: gradient echo; DWI: diffusion-weighted imaging; EPI: echo planar imaging; TE: echo time, TR: repetition time; FOV: field of view; MRI: magnetic resonance imaging

Parameter	T2	T1	DWI
Sequence type	3D SPACE	GRE	2D Reduced FOV EPI
TE (ms)	102	6.66	84
TR (ms)	1700	689	4500
Flip angle (degrees)	150	80	
FOV (mm)	220	220	190
Slice thickness (mm)	0.9	2	4
Resolution matrix	233 x 256	256 x 320	56 x 100

Our department MRI imaging protocol for prostate scanning includes two localiser scans to assess bladder and bowel compliance, followed by T2-weighted, T1-weighted and diffusion-weighted images of the pelvis (Table 2).

**Table 2:** Typical prostate radiotherapy MRI acquisition timeline HASTE: Half-Fourier Acquisition Single-shot Turbo spin Echo imaging; SPACE: Sampling Perfection with Application optimised Contrast using different flip angle Evolution; GRE: gradient echo; tra: transverse; MRI: magnetic resonance imaging

Sequence Type	Approx. Scan Time (min)	Function
Localiser	0.17	
HASTE Localiser	0.29	Positioning and review of bladder and rectal filling
Control 20 Slice rectum	0.30	Assessment of rectal filling (if required)
T2 SPACE tra	5.36	Prostate anatomy and tumour delineation, organ contouring. 3D isotropic sequence for urethral visualisation in sagittal plane
T1 GRE tra 2D	3.06	Gold fiducial identification (if required)
Diffusion Weighted Imaging	3.46	Delineation of dominant intraprostatic lesion (if required)

**Figure 1:**
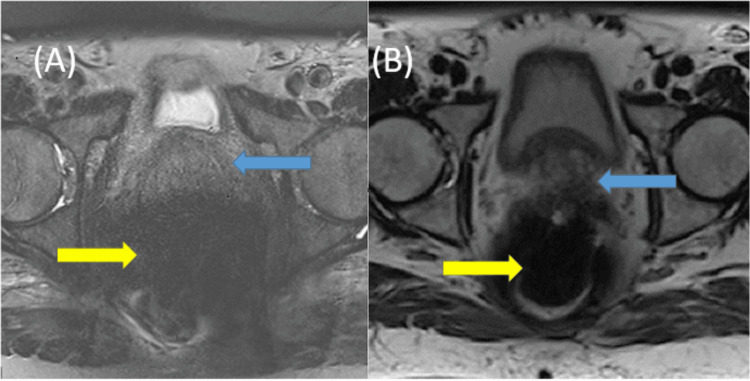
(A) Axial T2 SPACE and (B) axial T2 HASTE MRI sequences showing the prostate (blue arrow) in relation to susceptibility artefact (yellow arrow). SPACE: Sampling Perfection with Application optimised Contrast using different flip angle Evolution; HASTE: Half-Fourier Acquisition single-shot Turbo spin Echo imaging; MRI: magnetic resonance imaging

After acquiring the first localiser scan, MRI staff observed a large signal void with a prominent hypointense area in the small bowel, sigmoid colon and rectum (Figures 1A-1B), characterised as susceptibility artefact.

**Figure 2:**
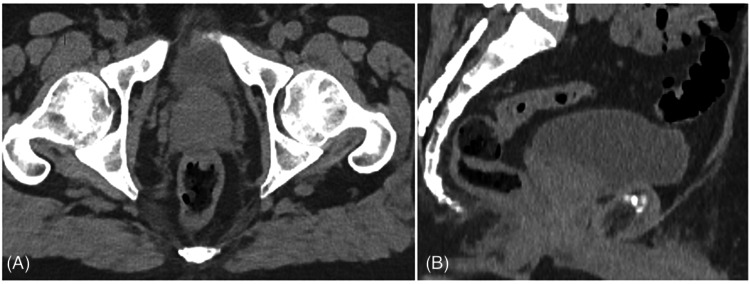
(A) Axial and (B) sagittal CT images confirming the absence of foreign bodies or high-density material.

Due to the unknown cause of the artefact, and to ensure patient safety was not potentially compromised, the scan was interrupted at this point and further inspection was conducted on the preceding CT scan to confirm no potential internal foreign objects were present. No foreign object or high-density artefact was observed on the CT scan (Figures 2A-2B) which prompted further investigation into the patient’s history.

The patient denied any foreign objects or implants but mentioned the only change to his regular dietary activity was commencing a daily supplement tablet which contained a minimum dry weight equivalent of 12.8g of curcumin (*Curcuma longa*), for a period of approximately six months to obtain anti-inflammatory and antioxidant benefits [5].

The radiographer on duty noted that the artefact observed appeared visually similar to susceptibility artefacts that can occur when scanning the stomachs of subjects who have ingested iron-fortified cereals and scanned immediately following ingestion [6].

A literary search discovered that curcumin may possess iron chelation characteristics [7,8] and it was hypothesised by the MRI staff that the patient’s curcumin supplements could be a potential explanation for the susceptibility artefact present in the small bowel and rectum.

The radiographer on duty confirmed it was safe for the patient to continue the exam and a T2-weighted sequence was acquired. The images confirmed a significant susceptibility artefact that was unable to be mitigated through sequence parameter adjustments and at this point, the remaining sequences of the examination were aborted.

After consulting with the RO and discussing the curcumin hypothesis, it was decided that due to the critical nature of the prostate gland and anterior rectal wall visualisation for the given diagnosis, the patient would be asked to cease curcumin supplements for one week and a repeat MRI scan would be attempted thereafter.

**Figure 3:**
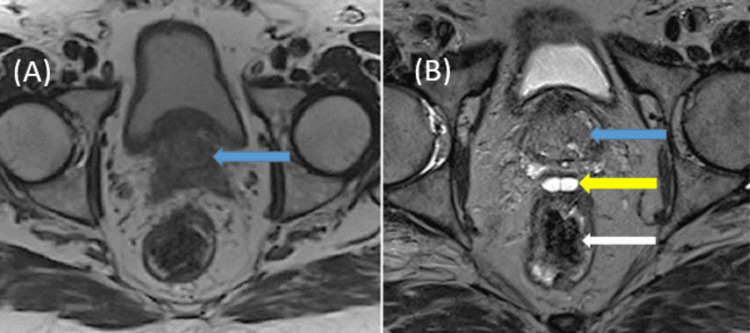
Repeat axial (A) and (B) T2 MRI sequences showing visualisation of the prostate (blue arrow), hydrogel barrier (yellow arrow) and rectum (white arrow) following cessation of curcumin supplements one week later.

The repeat CT and MRI scan was completed one week later, and a complete elimination of the susceptibility artefact had occurred (Figures 3A-3B). The RO was able to accurately distinguish between the required regions of interest and the patient proceeded to treatment as planned.

## Discussion

Initial images obtained during a pelvic MRI scan demonstrated significant susceptibility artefacts in the rectum and sigmoid colon, potentially attributed to curcumin supplements in the patient's gastrointestinal system. Curcumin (1,7-bis(4-hydroxy-3-methoxyphenyl)-1,6-heptadiene-3,5-dione or diferuloylmethane) is a naturally occurring polyphenol found in the rhizome of *C. longa* (commonly referred to as turmeric) and is known for its potential health benefits including anti-inflammatory, anti-viral, antimicrobial and antioxidant properties [9]. Curcumin is widely utilised as an easily accessible dietary supplement with a broad spectrum of biological applications, frequently consumed with the intention of enhancing overall health and well-being whilst being available for purchase without the need for a prescription [8]. However, it is essential to recognise that when curcumin is consumed by itself, it is also associated with inherent limitations that can affect its effectiveness in therapeutic applications. These limitations include poor absorption, limited bioavailability, rapid metabolism, and swift systemic elimination [10]. Furthermore, curcumin has been noted to possess chemical properties consistent with iron chelator activity [7] and maybe a useful management strategy for iron overload [11].

Spectroscopic shift techniques have demonstrated that iron binds to curcumin in solution [7], and curcumin’s mechanism of binding to iron follows the same binding mechanism as another common chelator, deferoxamine [12]. Deferoxamine as an iron chelator is primarily excreted through faeces [13], so it appeared a feasible hypothesis that if curcumin followed a similar path of primary excretion, the susceptibility artefact may have been caused by iron accumulation in the faeces.

The susceptibility artefact is visualised as either a bright (hyperintense) or dark (hypointense) region in the MR image, with the signal void appearing larger than the physical volume of the particle responsible for causing the artefact initially [14]. All materials possess some degree of intrinsic magnetic susceptibility, measured in parts per million (ppm), to quantify the tendency of a material to distort or interact with an applied magnetic field [15]. An object with differing magnetic susceptibility to its surroundings either augments or opposes the applied magnetic field and creates spatial variation in field strength surrounding the object, producing a susceptibility artefact.

Radiation therapy is widely recognised as a versatile and effective treatment for prostate cancer, utilising continually evolving technologies within radiation oncology to produce customisable treatment plans for the benefit of patients. In the treatment planning process, MRI continues to gain prominence as an integral imaging modality; as with other imaging techniques utilised in RTP, MRI acquisition can be influenced by a variety of factors that might result in suboptimal image quality [16,17]. Additionally, certain factors can increase the likelihood of artefact generation. In some cases, post-production artefact correction or reconstruction techniques can be employed to mitigate or substantially reduce artefact levels [17]. However, in our case, the significant size and location of the signal void meant such corrective measures were not able to be utilised to a clinically acceptable level.

During the MRI safety screening process, key focus areas include identifying any ferromagnetic or magnetically conductive objects located on the patient, and ensuring any potential medical implants are only exposed to the powerful magnetic field within the MRI scanning room if they are composed of materials that are non-conductive and are deemed either MRI safe or MRI conditional. In our patient's case, no medical history factors were disclosed that would have contraindicated MRI scanning, and the preceding CT imaging did not reveal the presence of any concerning high-density materials or metallic objects.

Of note, staff with extensive MRI expertise were present at the time of the scan. This expertise was a key reason that the artefact was identified as not being a safety concern for the patient, and that similarities to artefacts present in the abdomen of patients ingesting either iron folate supplements or iron-fortified cereals directly before an MRI exam were recognised. It was this field experience that led to the hypothesis as to the underlying cause of the artefact, along with a possible remedy. Without such experience and knowledge available on-site, it is likely the MRI scan would have been cancelled indefinitely. This would result in the RO relying solely on CT imaging for the radiotherapy contours, leaving some uncertainties in soft tissue delineation.

Ultimately, a robust knowledge of MRI principles was key in determining the cause and potential remedy for the artefact issue, allowing clear visualisation of both the anterior rectal wall and hydrogel barrier.

Considering the prevalence of dietary supplements such as curcumin and their potential to interact with MRI imaging, there may be a valid argument for incorporating a screening procedure for both routine and complimentary medications during radiation therapy work-up appointments. Radiation oncology patients frequently use complementary and alternative therapies and often do not discuss them to treating clinicians unless explicitly asked [18]. A more systemic screening approach could identify any iron-chelating substances and, in doing so, alterations to these medications could minimise the risk of unnecessary treatment delays and complications.

## Conclusions

The consumption of a daily curcumin supplement may have caused iron accumulation in our patient's gastrointestinal system, resulting in susceptibility artefacts on the pelvic MR images. Our patient required a repeat planning appointment to obtain images of satisfactory quality. This case reinforces the importance of conducting a rigorous pre-scanning safety questionnaire for all patients scheduled for an MRI scan, and the invaluable contribution knowledge and experience can provide in difficult or unusual scanning occurrences. While not definitively established by our case alone, it is worth considering the inclusion of a screening process for supplements known to possess chelation properties during the initial work-up appointments, especially when the quality of the MRI scan could potentially be compromised.
